# Prominent effects and neural correlates of visual crowding in a neurodegenerative disease population

**DOI:** 10.1093/brain/awu293

**Published:** 2014-10-28

**Authors:** Keir X. X. Yong, Timothy J. Shakespeare, Dave Cash, Susie M. D. Henley, Jennifer M. Nicholas, Gerard R. Ridgway, Hannah L. Golden, Elizabeth K. Warrington, Amelia M. Carton, Diego Kaski, Jonathan M. Schott, Jason D. Warren, Sebastian J. Crutch

**Affiliations:** 1 Dementia Research Centre, Department of Neurodegeneration, UCL Institute of Neurology, University College London, UK; 2 Centre for Medical Image Computing, University College London, UK; 3 University College London Hospitals NHS Foundation Trust, London, UK; 4 Department of Epidemiology and Population Health, London School of Hygiene and Tropical Medicine, London, England, UK; 5 Wellcome Trust Centre for Neuroimaging, UCL Institute of Neurology, Queen Square, London WC1N 3BG, UK; 6 Centre for Functional MRI of the Brain, University of Oxford, Oxford, UK; 7 Division of Brain Sciences, Imperial College London, Charing Cross Hospital, London, UK

**Keywords:** crowding, lateral masking, Alzheimer’s disease, posterior cortical atrophy, acquired dyslexia

## Abstract

Crowding is a breakdown in the ability to identify objects in clutter, and is a major constraint on object recognition. Crowding particularly impairs object perception in peripheral, amblyopic and possibly developing vision. Here we argue that crowding is also a critical factor limiting object perception in central vision of individuals with neurodegeneration of the occipital cortices. In the current study, individuals with posterior cortical atrophy (*n* = 26), typical Alzheimer’s disease (*n* = 17) and healthy control subjects (*n* = 14) completed centrally-presented tests of letter identification under six different flanking conditions (unflanked, and with letter, shape, number, same polarity and reverse polarity flankers) with two different target-flanker spacings (condensed, spaced). Patients with posterior cortical atrophy were significantly less accurate and slower to identify targets in the condensed than spaced condition even when the target letters were surrounded by flankers of a different category. Importantly, this spacing effect was observed for same, but not reverse, polarity flankers. The difference in accuracy between spaced and condensed stimuli was significantly associated with lower grey matter volume in the right collateral sulcus, in a region lying between the fusiform and lingual gyri. Detailed error analysis also revealed that similarity between the error response and the averaged target and flanker stimuli (but not individual target or flanker stimuli) was a significant predictor of error rate, more consistent with averaging than substitution accounts of crowding. Our findings suggest that crowding in posterior cortical atrophy can be regarded as a pre-attentive process that uses averaging to regularize the pathologically noisy representation of letter feature position in central vision. These results also help to clarify the cortical localization of feature integration components of crowding. More broadly, we suggest that posterior cortical atrophy provides a neurodegenerative disease model for exploring the basis of crowding. These data have significant implications for patients with, or who will go on to develop, dementia-related visual impairment, in whom acquired excessive crowding likely contributes to deficits in word, object, face and scene perception.

## Abstract (precis)

Visual crowding is a perceptual phenomenon whereby recognition of a stimulus is disrupted by the presence of flanker stimuli. Yong *et al.* observe excessive crowding in individuals with a neurodegenerative condition (posterior cortical atrophy) and identify associations between prominent crowding and lower grey matter volume in the right collateral sulcus.

## Introduction

Crowding is a form of inhibitory interaction that is present in spatial vision, involving the diminishing effect of nearby stimuli (‘flankers’) on the identification of a target stimulus (Levi, 2008). The occurrence of crowding when target stimuli and flankers are separately presented to different eyes indicates a cortical locus (Flom *et al.*, 1963; Tripathy and Levi, 1994). Crowding is dependent on eccentricity (distance from fixation), which determines the critical spacing between target and flankers (Pelli *et al.*, 2007). The critical spacing is the distance at which flankers inhibit identification of the target stimulus, and has been roughly localized as being half the eccentricity of the target from fixation in peripheral vision (Bouma, 1970). While crowding is independent of stimulus type, font and contrast (Tripathy and Cavanagh, 2002; Pelli *et al.*, 2004; Pelli and Tillman, 2008), crowding effects diminish with target and flanker stimuli of opposite polarity (Kooi *et al.*, 1994; Chakravarthi and Cavanagh, 2007) and are exacerbated by increasing visual similarity between target and flanker stimuli (Kooi *et al.*, 1994; Bernard and Chung, 2011). Beyond being the crucial limiting factor in normal peripheral vision, crowding poses particular problems for certain patient populations, such as individuals with amblyopia, macular degeneration or apperceptive agnosia (Song *et al.*, 2014).

Although some theories of crowding suggest that it may arise from poor resolution of attention (Intriligator and Cavanagh, 2001) or unfocussed higher-order spatial attention (Strasburger, 2005), crowding tends to be considered a pre-attentive process related to the interaction between simple visual features. Three main classes of theories have been proposed: the first, a classic lateral masking perspective, associates crowding with low level masking—a consequence of competition between a finite number of feature detectors (Townsend *et al.*, 1971; Wolford and Chambers, 1984), which may occur at the level of the retina, lateral geniculate nucleus, or the primary visual cortex (V1) (Chakravarthi and Cavanagh, 2007). Second, substitution accounts propose that flanker features (or whole flankers) are mistakenly intermixed with the target; this substitution is most commonly attributed to noisy processing of the position of flanker and targets and/or their features (Wolford, 1975; Krumhansl and Thomas, 1977; Nandy and Tjan, 2007) which may arise as a consequence of larger receptive fields (Dayan and Solomon, 2010). Third, averaging accounts suggest that crowding arises as a problem of excessive feature integration: when cells responsible for pooling/averaging information over a large area encounter flankers, information about the flanker stimuli is assimilated with information about the target stimulus (Levi *et al.*, 2002; Pelli *et al.*, 2004; Greenwood *et al.*, 2009, 2010).

Of these three classes of theory, the first (lateral masking) has been distinguished from the second and third (substitution and averaging) with the suggestion that crowding limits identification of target stimuli, whereas lateral masking limits both identification and detection (Parkes *et al.*, 2001). Levi’s (2008) review of crowding suggests a growing consensus around a two-stage model of crowding encompassing both detection of simple features, possibly in V1, and integration of features downstream from V1. Support for this multi-stage view comes from a recent functional MRI study of the neural correlates of crowding in healthy individuals. Using a change-detection paradigm involving oriented Gabor patches, crowding was found to influence neural responses throughout cortical visual areas V1–V4, an effect that increased in strength from early to late visual areas (Anderson *et al.*, 2012). These findings differ from earlier studies that did not identify an effect of crowding upon V1 responses (Arman *et al.*, 2006; Fang *et al.*, 2008; Bi *et al.*, 2009) and argue against depictions of crowding as a later-stage process only arising following feature detection.

The overwhelming majority of studies of crowding have been conducted in normal peripheral or amblyopic vision. Crowding purportedly occurs in normal foveal vision but only over tiny distances (a few minutes of arc; Bouma, 1970) so most conventionally printed text (like the 1.2° letters used in the current study) would have to overlap to induce crowding, thus making it difficult to distinguish effects of crowding from ordinary masking (Martelli *et al.*, 2005). However, two recent case reports have suggested that individuals with the neurodegenerative syndrome posterior cortical atrophy (PCA) exhibit letter identification deficits in central vision that may be consistent with acquired excessive crowding (Crutch and Warrington, 2007, 2009). PCA is characterized by progressive visual impairment and tissue loss in posterior brain regions; patients tend to have underlying Alzheimer’s disease pathology, but pathological changes are more concentrated in occipital, parietal and temporo-occipital rather than medial temporal regions relative to patients with typical amnestic Alzheimer’s disease (Hof *et al.*, 1997). The two case reports assessing flanked letter identification abilities in PCA identified beneficial effects of increased spacing on flanked letter identification, interactions between letter spacing and letter confusability and an ameliorating effect of reverse polarity flankers; all of these effects are characteristic of crowding. However, there have been no group investigations of excessive crowding in PCA or any other neurodegenerative condition. The incidence of crowding in PCA and typical Alzheimer’s disease remains unknown, as does the extent to which crowding may contribute to more recognized deficits of object and space perception in these conditions.

This study aimed to examine crowding in the context of neurodegenerative disease, addressing the question of why individuals with PCA show excessive crowding in central vision. First, we evaluated whether the characteristics of flanked letter identification performance in PCA are consistent with the properties of crowding, and estimated the prevalence of crowding deficits in PCA. Second, given the attenuating effects of increased spacing and reverse polarity on crowding in normal peripheral vision, we tested whether manipulation of these variables would lead to facilitation of letter identification in PCA. Third, we evaluated whether the letter identification errors observed in PCA were more consistent with averaging or substitution accounts of crowding, by testing how well averaged or additive models of targets and/or flankers predicted error identity and frequency. Finally, we established the structural neural correlates of crowding in PCA, and considered their correspondence to previous functional imaging studies of crowding in healthy individuals.

## Materials and methods

### Participants

The study participants were 26 patients with PCA, 17 patients with typical Alzheimer’s disease and 14 healthy control subjects. The patients with PCA all fulfilled clinical criteria for a diagnosis of posterior cortical atrophy (Mendez *et al.*, 2002; Tang-Wai *et al.*, 2004; McMonagle *et al.*, 2006). Patients with PCA and patients with typical Alzheimer’s disease fulfilled research criteria for probable Alzheimer’s disease (Dubois *et al.*, 2007, 2010). Molecular pathology (^18^ F amyloid imaging performed as part of another investigation or CSF referred as part of their diagnostic work-up) was available for 7/26 patients with PCA and 11/17 patients with typical Alzheimer’s disease (Table 1); results for all typical Alzheimer’s disease patients and 6/7 patients with PCA were consistent or borderline consistent with Alzheimer’s disease pathology (positive amyloid scan on standard visual rating or CSF amyloid-β_1-42_ ≤ 450 pg/ml and/or tau/amyloid-β ratio >1). The healthy control subjects were matched to the PCA and typical Alzheimer’s disease groups on age and years of education, with the PCA and typical Alzheimer’s disease participants additionally matched for disease duration and Mini Mental State Examination score (MMSE; Table 2).

**Table 1:** Molecular pathology data for patients with PCA and typical Alzheimer’s disease

Diagnosis	Amyloid ^18^ F imaging	CSF total tau pg/ml	CSF amyoid-β_1-42 _ pg/ml	CSF tau:amyloid-β ratio	Interpretation
PCA	Not available	310	488	0.64	−
PCA	Not available	931	625	1.49	+
PCA	positive	1072	126	8.51	++
PCA	Not available	151	147	1.03	+
PCA	positive	Not available	Not available	Not available	++
PCA	positive	1082	365	2.96	++
PCA	positive	Not available	Not available	Not available	++
tAD	Not available	289	280	1.03	+
tAD	Not available	757	285	2.66	++
tAD	Not available	940	348	2.70	++
tAD	Not available	952	195	4.88	++
tAD	Not available	977	322	3.03	++
tAD	Not available	625	277	2.26	++
tAD	Not available	>1200	313	>3.83	++
tAD	Not available	913	191	4.78	++
tAD	Not available	>1200	217	>5.52	++
tAD	Not available	1099	195	5.64	++
tAD	Not available	850	362	2.35	++

tAD = typical Alzheimer’s disease.

Where results do not support Alzheimer’s disease pathology (−), are borderline consistent with Alzheimer’s disease pathology (+) and are >85% specific for Alzheimer’s disease pathology (++). Amyloid (florbetapir) PET scans were received as part of another investigation.

**Table 2:** Demographic information and neuropsychological scores of patients with PCA and typical Alzheimer’s disease

**Demographic information**		**PCA (*n* = 26)**	**tAD (*n* = 7)**	**Control (*n* = 14)**	

Gender (male:female)		10/16	12/5	5/9	
Age (mean years ± SD)		61.4 ± 7.7	65.0 ± 5.1	62.7 ± 5.0	
Education level (mean years ± SD)		14.6 ± 2.3	14.9 ± 2.4	16.1 ± 2.4	
Disease duration (mean years ± SD)		4.7 ± 3.1	5.0 ± 1.7	–	
MMSE^a^ (mean/30 ± SD)		17.7 ± 5.0	17.5 ± 4.9	–	

**Neuropsychology test**	**Max score**	**Raw score**			**Norms/comment**
		**PCA**	**tAD**	**Difference *P*-value**	
**Background Neuropsychology**					
Short Recognition Memory Test^b^ for words* (joint auditory/visual presentation)	25	19.5 ± 3.7	14.7 ± 1.5	*<0.0001*	PCA: 5^th^–10^th^ percentile, tAD: ∼<5^th^ percentile (cut-off: 19)
Short Recognition Memory Test for faces*	25	17.8 ± 4.0	16.8 ± 3.0	*>0.3*	Both ∼<5^th^ percentile (cut-off: 18)
				
Concrete Synonyms test^c^	25	20.0 ± 3.7	20.9 ± 2.5	*>0.4*	Both 10^th^–25^th^ percentile
Naming (verbal description)	20	11.4 ± 6.6	13.7 ± 6.4	*>0.2*	Both ∼<5^th^ percentile (cut-off: 15)
Cognitive estimates^d^ (error score)	30	14.6 ± 7.5	10.6 ± 5.0	*0.074*	Both ∼<1^st^ percentile (cut-off: 9)
Calculation (GDA^e^)*	24	1.6 ± 2.9	4.9 ± 5.3	*<0.05*	PCA: ∼<5^th^ percentile, tAD:5^th^-25^th^ percentile
Spelling (GDST^f^- Set B, first 20 items)*	20	8.9 ± 6.5	10.8 ± 5.6	*>0.3*	Both 10^th^–25^th^ percentile
Gesture production test^g^	15	12.7 ± 3.4	14.1 ± 1.4	*>0.1*	–
Digit span (forwards)	12	6.0 ± 2.6	6.1 ± 1.4	*>0.8*	Both 25^th^–50^th^ percentile
Max forwards	8	5.6 ± 1.8	5.5 ± 0.8	*>0.9*	–
Digit span (backwards)	12	2.6 ± 1.7	3.6 ± 1.9	*0.078*	Both 5^th^–10^th^ percentile
Max backwards	7	2.3 ± 1.3	3.3 ± 1.1	*<0.05*	–
CORVIST^h^ reading test	16	13.8 ± 3.0	15.7 ± 0.8	*<0.05*	–
Spatial attention^i^:					
Small words	48	39.9 ± 10.7	47.2 ± 2.2	*<0.0005*	–
Large words	48	37.8 ± 12.5	47.1 ± 1.6	*<0.0005*	–
**Visual assessment**					
*Early visual processing*					
Visual acuity (CORVIST): Snellen	6/9	(median 6/9)	(median 6/9)		Normal acuity
Figure-ground discrimination (VOSP^j^)	20	16.3 ± 3.0	18.6 ± 1.3	*<0.01*	PCA: ∼<5^th^ percentile, tAD: 5^th^–10^th^ percentile
Shape discrimination^k^	20	12.6 ± 3.9	17.2 ± 3.2	*<0.0005*	Healthy controls do not make any errors
Hue discrimination (CORVIST)	4	2.6 ± 1.1	3.0 ± 1.3	*>0.3*	–
					
*Visuoperceptual processing*					
Object Decision (VOSP)*	20	10.0 ± 4.1	15.9 ± 2.4	*<0.0001*	PCA: ∼<5^th^ percentile, tAD: 10^th^–25^th^ percentile
Fragmented letters (VOSP)	20	2.9 ± 3.9	13.5 ± 6.6	*<0.0001*	Both ∼<5^th^ percentile (cut-off: 16)
Unusual and usual views:^l^					
Unusual	20	6.6 ± 6.8	9.9 ± 5.1	*>0.1*	Both ∼<1^st^ percentile (cut-off: 12)
Usual	20	8.4 ± 5.5	16.5 ± 4.0	*<0.0001*	Both ∼<1^st^ percentile (cut-off: 18)
					
*Visuospatial processing*					
Number location (VOSP)*	10	1.8 ± 2.5	5.7 ± 3.8	*<0.005*	Both ∼<5^th^ percentile (cut-off: 6)
Dot counting (VOSP)	10	3.4 ± 3.2	8.1 ± 3.1	*<0.0001*	Both ∼<5^th^ percentile
A Cancellation^m^: Completion time	90 s	79.5 s ± 17.4	36.3 s ± 15.7	*<0.0001*	Both ∼<5^th^ percentile (cut-off: 32 s)
A Cancellation^m^: Number of letters missed	19	6.6 ± 5.1	0.53 ± 1.1	*<0.0005*	–

*Behavioural screening tests supportive of PCA diagnosis; tAD = typical Alzheimer’s disease.

^a^Folstein *et al.*, 1975.

^b^Warrington, 1996.

^c^Warrington *et al.*, 1998.

^d^Shallice and Evans, 1978.

^e^Graded Difficulty Arithmetic test (GDA) (Jackson and Warrington, 1986).

^f^Graded Difficulty Spelling Test (GDST) (Baxter and Warrington, 1994).

^g^Crutch (unpublished).

^h^Cortical Visual Screening Test (CORVIST) (James *et al.*, 2001).

^i^Perceptual corpus (Yong *et al.*, 2014).

^j^Visual Object and Space Perception Battery (VOSP) (Warrington and James, 1991).

^k^Efron (1968): oblong edge ratio 1:1.20.

^l^Warrington and James (1988).

^m^Willison and Warrington (1992).

### Background neuropsychology and visual assessment

Patients with PCA and typical Alzheimer’s disease were administered a battery of neuropsychological tests assessing memory, language, arithmetic, spelling, reading and early visual, visuoperceptual and visuospatial processing (Table 2).

### Crowding assessment

**Figure 1:**
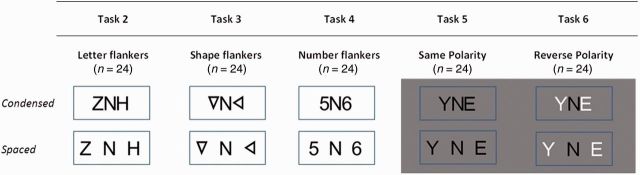
Target/flanker arrays used in Tasks 2–6 under different spacing conditions.

All participants were requested to name target stimuli (uppercase letters excluding I, J, O, Q, W and X) under the following conditions (example stimuli are shown in Fig. 1).

#### Task 1: Unflanked letter identification

The target stimuli (*n* = 20) were alphabetic items presented in isolation. Letters were presented in random order for 6000 ms in a fixation box (3.2° in width, 2.9° in height) at the centre of the screen.

In each of the following tasks (Tasks 2–6), target letter identification was probed under two spatial conditions, condensed and spaced.

#### Task 2: Letter flankers

Target letters (*n* = 24) were flanked on each side by a letter, forming a 3-letter non-word combination.

#### Task 3: Shape flankers

Target letters (*n* = 24) were flanked on each side by a triangle presented at different orientations. Triangles were of equal height and line thickness to target letters.

#### Task 4: Number flankers

Target letters (*n* = 24) were flanked on each side by an Arabic numeral, chosen from a range between 2 and 9.

#### Task 5: Same-polarity flankers

Target letters (*n* = 24) were flanked on each side by black letters; presentation was as Task 2 except that items were presented on a grey background to match Task 6 (see below).

#### Task 6: Reverse-polarity flankers

Target black letters (*n* = 24) were flanked on each side by white letters, all presented on a grey background.

The edge-to-edge distance between the target letter and flankers was 0.1° of visual angle in the condensed condition and 1.0° in the spaced condition at a viewing distance of 50 cm; the height of stimuli (10.5 mm) corresponded to a visual angle of 1.20°. Participants were given one prompt for each trial where they named the flanker (‘Is that the letter in the middle?’); this prompt was intended to limit errors resulting from visual disorientation. The same combination of flankers was used for each target letter under both spatial conditions within each flanker condition. Alphabetic items occurred with equal frequency within each task. The stimuli were presented in blocks of six items, with blocks being administered in an ABBA design. All flanked stimuli were presented in the centre of the screen within a fixation box (6.4° in width, 2.9° in height). All 26 patients with PCA completed Tasks 1, 2 and 4; 24 completed Task 3; and 22 completed Tasks 5 and 6. Naming latencies were manually determined from the onset of each letter using Audacity (http://audacity.sourceforge.net).

### Data analysis

#### Behavioural covariates

Tests of early visual, visuoperceptual and visuospatial processing (Table 2) were transformed and averaged to form composite scores for each visual domain. Raw scores were transformed into a standardized range (0–100) in which 0 and 100 corresponded to the minimum and maximum score achieved by any patient (PCA or typical Alzheimer’s disease), respectively. The following raw scores were also transformed into a standardized range for the PCA versus typical Alzheimer’s disease regression analysis: unflanked letter identification, digit span (backwards), A cancellation time, a measure of peripheral spatial attention (discrepancy in accuracy for a subset of words selected from the perceptual corpus (Yong *et al.*, 2014) at two levels of size (large: *n* = 48; letter height 2°; small: *n* = 48; letter height 0.5°) words were matched for frequency, age of acquisition and concreteness and were administered in the same testing session as the crowding assessment. MMSE and disease duration were also used as behavioural covariates.

#### Crowding indices for voxel-based morphometry analysis

Scores on shape and number flankers (Tasks 3 and 4) were used as crowding indices, based on the rationale that errors with non-letter flankers were less likely to reflect attentional or executive deficits. Indices were based on raw score differences:

- Spacing (shape): difference in accuracy between spacing conditions (spaced-condensed) in Task 3 (shape flankers);
- Spacing (number): difference in accuracy between spacing conditions (spaced-condensed) in Task 4 (number flankers);
- Spacing (shapes/numbers): difference in accuracy between spacing conditions (spaced-condensed) in Tasks 3 and 4 combined;
- Polarity: difference in accuracy between Tasks 5 and 6 (reverse-same polarity);
- Polarity (condensed): difference in accuracy between Tasks 5 and 6 (condensed condition only).

#### Data trimming

Latency data for erroneous responses, responses where participants had become overtly distracted from the task, or responses following prompts were removed from the analysis [total *n* = 1397 (21.6%)]. Latency data greater than two standard deviations from the mean of each participant were considered outliers and removed [total *n* = 442 (8.73%)]. Prior to latency regression analysis, latency data were transformed using inverse transformation due to non-normal distribution of residuals. Naming latency data were only analysed for participants who made no errors or did not make enough errors to produce significant effects of spacing or flanker type on naming speed using logistic regression or chi-squared tests at the individual level (Crowding assessment 1: PCA: *n* = 12, MMSE = 18.8, disease duration = 3.9 years; Crowding assessment 2: PCA: *n* = 9, MMSE = 20.0, disease duration = 3.0 years; all typical Alzheimer’s disease and control participants).

#### Statistical analysis

**Figure 2:**
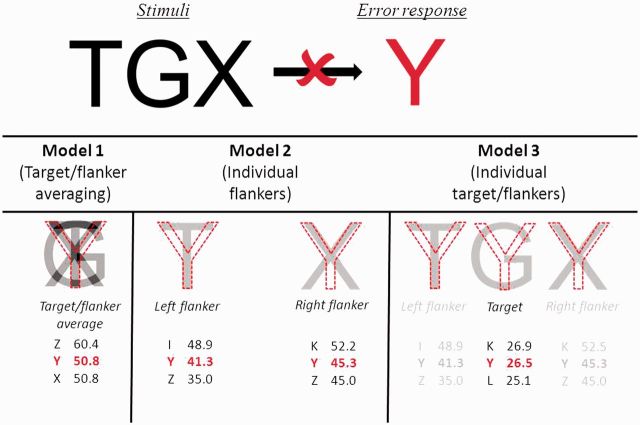
Three models assessing whether the identity of error responses could be predicted on the basis of the similarity between that error response and the averaged (Model 1) or individual (Models 2 and 3) overlap with flanker and/or target stimuli. The current example involves the stimulus ‘TGX’ yielding an error response of ‘Y’. Values in red refer to the visual similarity of the error response (higher values represent greater similarity) generated from the overlap in pixels with each item (averaged target/flanker, or individual target/flankers). Values in black refer to responses which have similarity values closest to the error response.

Analysis of accuracy and latency data was conducted using logistic and linear models, respectively; both models used robust standard errors to account for clustering by participant. Spacing, flanker type, the interaction between spacing and flanker, and their interactions with participant group were the variables of interest, while the linear model also included accuracy as a covariate. Between patient group (PCA versus typical Alzheimer’s disease) regression analyses used the same logistic and linear models but also included diagnosis and one of the behavioural covariates listed above. Differences between PCA and typical Alzheimer’s disease groups were calculated using a Wilcoxon rank-sum test and differences within groups were calculated using a Wilcoxon signed-rank test. Three models were used to determine whether similarity between target and/or flanker stimuli and error responses predicted error rates (Fig. 2):

- Model 1 (target/flanker averaging): similarity values generated from averaged target and flanker stimuli;
- Model 2 (individual flankers): similarity values independently generated from each flanker; both were included in the model; and
- Model 3 (individual flankers and target): identical to Model 2 except that similarity values independently generated from the target were also included.

Similarity values were derived from the overlap in pixels between target and/or flanker stimuli and error responses. All models were logistic mixed-effects models with random-effects of participant and error response and fixed effects of the similarity values for each of the three models detailed above.

### Neuroimaging data

T_1_-weighted volumetric magnetic resonance images were acquired on a Siemens Trio TIM 3T scanner for 20 patients with PCA (see Supplementary material for details on image acquisition). Voxel-based morphometry was performed using SPM8 software (Statistical Parametric Mapping, Version 8; http://www.fil.ion.ucl.ac.uk/spm) running on MATLAB R2012a (http://www.mathworks.com). Images were rigidly orientated to standard Montreal Neurological Institute (MNI) space using the ‘New segment’ function in SPM8. Rigidly-orientated scans were segmented into grey matter, white matter and CSF. The Dartel toolbox (Ashburner, 2007) was used to perform spatial normalization, first aligning grey matter and white matter segmentations to their group-wise average (Ashburner and Friston, 2009), then combining this transformation with an affine mapping to MNI space. Normalized segmentations were modulated to preserve native-space tissue volumes and smoothed with a 6 mm full-width at half-maximum Gaussian kernel. A group-wise custom template in MNI space was created by arithmetically averaging the Dartel-normalized bias-corrected MP-RAGE images of all 20 PCA participants. Associations between regional grey matter volume and indices of crowding were assessed using voxel-wise linear regression models in SPM8. Total intracranial volume, age, gender and MMSE score were included as covariates. A whole-brain grey matter mask was defined to include voxels for which the intensity was >0.1 in at least 80% of the images; this has been shown to be appropriate for participants with greater atrophy (Ridgway *et al.*, 2009). A region of interest mask was defined for the occipital lobe; this was created using the Hammers atlas, which was warped to the custom template. Associations between grey matter volume and behavioural performance were assessed over the whole brain and within the occipital region of interest specified by previous anatomical hypotheses. A voxel-wise statistical threshold of *P* < 0.05, family-wise error corrected for multiple comparisons was applied in all analyses. Statistical parametric maps were overlaid on the custom template.

## Results

### Background neuropsychology and visual assessment

Mean scores for the PCA and typical Alzheimer’s disease groups and an estimate of their performance relative to normative data sets appropriate for the mean age of each group are shown in Table 2. On tasks without a visual component, the performance of the PCA group was mostly equivalent to (Concrete Synonyms, Naming, Digit Span forwards) or better than (Short Recognition Memory Test: words) that of the typical Alzheimer’s disease group; however, performance on measures of reading, visual processing and some non-visual tasks associated with parietal function (Calculation, Digit Span backwards) was worse than in the typical Alzheimer’s disease group.

#### Visual status

Of the 26 patients with PCA, a measure of visual acuity (Cortical Vision Screening Test) administered to both eyes identified normal visual acuity (Snellen) in 18 patients (69.2%) had a visual acuity of 6/9–6/12 (Snellen chart); four patients (15.4%) demonstrated a visual acuity of 6/18, one patient demonstrated a visual acuity of 6/24 and one patient demonstrated a visual acuity of 6/36. This acuity measure could not be effectively administered to the remaining two patients with PCA due to profound visuospatial impairment. All patients with typical Alzheimer’s disease demonstrated normal corrected visual acuity on this measure.

Eleven patients with PCA were assessed by ophthalmologist or neuro-ophthalmologist; thorough examination found no evidence of an ophthalmologic condition and/or attributed any loss of vision to a disorder of the visual cortex in eight patients. One patient had cataracts but demonstrated normal visual acuity (6/6 in each eye) and results were not available for the remaining two patients.

### Crowding assessment 1: flanker and spacing effects

The mean and standard deviation percentage accuracy and naming latency results for each group on Tasks 1–6, plus group comparisons, are shown in Table 3.

**Table 3:** Comparisons between PCA and typical Alzheimer’s disease group accuracy and latency data

*Task*	*N*	**Naming accuracy (%)**			**Group comparisons (*p* values)**		
		**Groups**					
		PCA	tAD	Controls	PCA vs tAD	PCA vs controls	tAD vs controls
1. Unflanked letter identification	20	99.8 ± 0.2	100 ± 0	100 ± 0	p > .4	p > .4	–
2. Letter flankers	24	75.8 ± 25.1	99.3 ± 1.6	100 ± 0	p < .0001	p < .0001	p > .1
3. Shape flankers	24	83.5 ± 18.6	99.7 ± 1.0	100 ± 0	p < .0005	p < .0005	p > .3
4. Number flankers	24	83.6 ± 23.5	99.7 ± 1.0	100 ± 0	p < .0005	p < .0005	p > .3
5. Same polarity letter flankers	24	78.8 ± 22.5	98.5 ± 2.9	100 ± 0	p < .001	p < .0005	p = .065
6. Reverse polarity letter flankers	24	86.5 ± 15.6	99.3 ± 2.2	100 ± 0	p < .001	p < .0005	p > .2
**Summary data**							
Total (Tasks 2–4)	72	81.3 ± 19.7	99.6 ± 1.1	100 ± 0	p < .0001	p < .0001	p > .1
Total condensed (Tasks 2–4)	36	72.0 ± 26.7	99.7 ± 0.9	100 ± 0	p < .0001	p < .0001	p > .2
Total spaced (Tasks 2–4)	36	90.0 ± 16.0	99.5 ± 1.5	100 ± 0	p < .001	p < .001	p > .2
Total (Tasks 5–6)	48	82.7 ± 18.3	98.9 ± 2.2	100 ± 0	p < .005	p < .0005	p < .05

*Task*	*N*	**Naming latency (ms)**			**Group comparisons (*p* values)**		
		**Groups**					
		PCA	tAD	Controls	PCA vs tAD	PCA vs controls	tAD vs controls
1. Unflanked letter identification	20	785 ± 315	583 ± 95	497 ± 53	p = .056	p < .005	p < .05
2. Letter flankers	24	2726 ± 3196	634 ± 128	570 ± 113	p < .0001	p < .0001	p > .2
3. Shape flankers	24	1631 ± 867	546 ± 86	538 ± 95	p < .0001	p < .0001	p > .9
4. Number flankers	24	2072 ± 1842	548 ± 92	469 ± 119	p < .0005	p < .0005	p < .05
5. Same polarity letter flankers	24	1836 ± 1167	609 ± 128	518 ± 124	p < .001	p < .0005	p = .063
6. Reverse polarity letter flankers	24	2133 ± 1635	591 ± 91	506 ± 131	p < .005	p < .001	p < .05
**Summary data**							
Total (Tasks 2–4)	72	2054 ± 1694	573 ± 94	524 ± 100	p < .0001	p < .0001	p > .2
Total condensed (Tasks 2–4)	36	2510 ± 2481	591 ± 98	537 ± 100	p < .0001	p < .0001	p > .1
Total spaced (Tasks 2–4)	36	1659 ± 1073	555 ± 95	515 ± 101	p < .0005	p < .0005	p > .3
Total (Tasks 5–6)	48	2004 ± 140	597 ± 104	512 ± 126	p < .001	p < .0005	p = .090

#### Task 1: Unflanked letter identification

There was no significant difference in unflanked letter identification accuracy between the PCA and typical Alzheimer’s disease or control groups. One PCA patient made one error, while the typical Alzheimer’s disease and control groups did not make any errors. There was a trend towards the PCA group having longer naming latencies than the typical Alzheimer’s disease group. Both PCA and typical Alzheimer’s disease groups were slower than the control group.

#### Tasks 2–4: Letter, shape and number flankers

**Figure 3:**
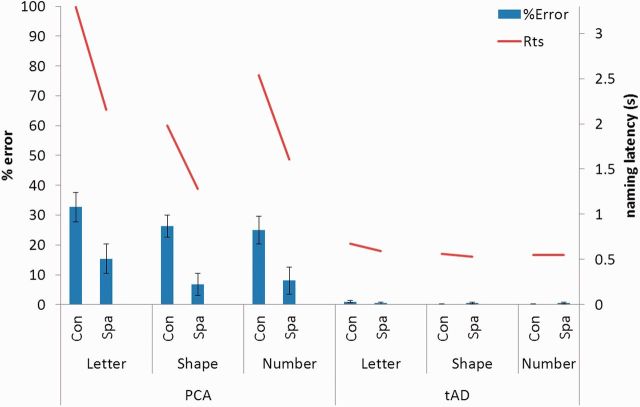
Accuracy and naming latency data for the PCA and typical Alzheimer’s disease (tAD) group for letter, shape and number flankers in both spatial conditions. Rts = reaction time; Con = condensed; Spa = spaced. Error bars show standard deviation.

A summary of the PCA and typical Alzheimer’s disease accuracy and naming latency data is shown in Fig. 3. The patients with PCA were consistently worse than both typical Alzheimer’s disease patients and controls in terms of both naming accuracy and latency on Tasks 2–4. The accuracy of patients with typical Alzheimer’s disease was not significantly different from that of control subjects on any task, but they were slower on Task 4.

#### Tasks 2–4: Effects of spacing

Patients with PCA (*n* = 26) showed significantly poorer accuracy for target letter identification in the condensed than spaced condition (z = 7.81, *P* < 0.001). Analysis of latency data (*n* = 10) also identified longer naming latencies in the condensed condition (*t* = 3.33, *P* < 0.01). At the individual patient level, 18/26 (69.2%) showed a spacing effect in accuracy and/or latency: all but three of those (15/26: 57.7%) showed this spacing effect even when analysis was restricted to non-letter flankers. Similar proportions of patients with PCA with no clinically identifiable ophthalmological impairment showed a spacing effect in accuracy and/or latency (6/8: 75.0%) and a spacing effect when analysis was restricted to non-letter flankers (4/8: 50.0%). Of the patients with PCA with impaired visual acuities (visual acuity <6/12), 3/6 (50.0%) showed a spacing effect on their accuracy and/or latency, with 1/6 (16.7%) showing a spacing effect when analysis was restricted to non-letter flankers. Patients with typical Alzheimer’s disease did not demonstrate an effect of spacing (*P* > 0.5) on accuracy but latencies were significantly longer in the condensed condition (t = 4.73, *P* < 0.001). Controls made no errors, but did show longer latencies in the condensed condition (t = 2.89, *P* < 0.05). Analysis of combined accuracy data from the PCA and typical Alzheimer’s disease groups revealed a significant interaction between diagnosis and spacing (z = −2.77, *P* < 0.01), with patients with PCA showing a greater spacing effect; no such interaction was found for naming latencies (*P* > 0.1). Analysis of combined latency data for the typical Alzheimer’s disease and control groups found no evidence of an interaction between diagnosis and spacing (*P* > 0.4).

#### Tasks 2–4: Effects of flanker category

Patients with PCA showed significantly poorer accuracy for letters relative to other flanker categories (versus shapes: z = 2.68, *P* < 0.01; versus numbers: z = 2.61, *P* < 0.01). However, this between-category difference only held in the spaced condition (versus shapes: z = 3.65, *P* < 0.001; versus numbers: z = 3.55, *P* < 0.001); in the condensed condition, there was no significant difference between letters and other flankers (versus shapes/numbers: *P* > 0.1). In contrast, both typical Alzheimer’s disease and control groups showed longer latencies for letter flankers in both spaced and condensed conditions. Patients with PCA showed significantly slower naming latencies for letters relative to other flanker categories (versus shapes: t = 3.74, *P* < 0.005; versus numbers: t = 2.60, *P* < 0.05); this between-category difference was consistent across spacing conditions. Patients with typical Alzheimer’s disease did not show poorer accuracy for letters (versus shapes/numbers: *P* > 0.1) but did show longer latencies (versus shapes: t = 6.13, *P* < 0.001; versus numbers: t = 5.13, *P* < 0.001). Controls made no errors, but showed longer latencies for letter flankers (versus shapes: t = 2.59, *P* < 0.05; versus numbers: t = 3.55, *P* < 0.005). Analysis of combined accuracy or latency data from the PCA and typical Alzheimer’s disease groups did not find significant interactions between diagnosis and flanker type. Analysis of combined latency data for the typical Alzheimer’s disease and control groups revealed a significant interaction between diagnosis and flanker type, with patients with typical Alzheimer’s disease slower in the letter relative to the shape (t = 2.38, *P* < 0.05) but not the number conditions (t = −1.90, *P* =0 .067).

#### *Post hoc* analysis of covariates

None of the behavioural covariates [early visual, visuoperceptual and visuospatial processing, unflanked letter identification, Digit Span (backwards), A Cancellation time, a measure of peripheral spatial attention, MMSE or disease duration] could account for the spacing effect within the PCA or typical Alzheimer’s disease groups. None of the covariates could account for the overall group difference in naming accuracy between the PCA and typical Alzheimer’s disease groups. Similarly, none of the covariates could account for group differences when considering the condensed condition alone, however, visuoperceptual (z = 2.13, *P* < 0.05; PCA versus typical Alzheimer’s disease: *P* > 0.2) and visuospatial (z = 3.60, *P* < 0.001; PCA versus typical Alzheimer’s disease: *P* > 0.1) function did account for the group difference in the spaced condition. Linear regression analysis found that none of the covariates could account for the group difference between the PCA and typical Alzheimer’s disease groups for naming latency overall or in either spacing condition.

#### *Post hoc* eyetracking analysis

**Figure 4:**
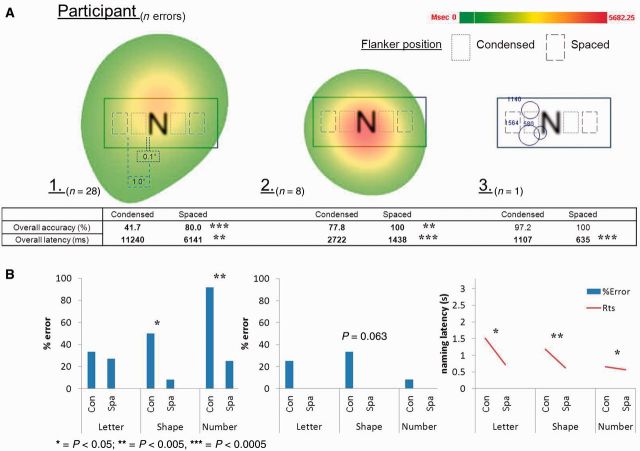
(**A**) Eyetracking data for trials where three patients with PCA made errors naming flanked letter stimuli across different conditions of spacing/flanker (Tasks 2–4). Heat maps for Participants 1 and 2 show total maximum fixation duration within an area averaged across trials; individual fixation duration is shown for Participant 3. (**B**) Accuracy and naming latency data for letter, shape and number flankers in both spatial conditions. Rts = reaction time; Con = condensed; Spa = spaced.

Additional *post hoc* eyetracking data were gathered on three individuals with PCA (one of whom took part in the original crowding assessments) to confirm that patients were fixating centrally when making errors naming flanked target stimuli. These three patients demonstrated spacing effects on their naming accuracy and/or latency across flanker conditions. A head-mounted Eyelink II system was used to record gaze location at 250 Hz, with fixations and saccades parsed at standard velocity and acceleration thresholds (30°/s and 8000°/s^2^). Fixation data suggest that erroneous responses were made when participants were using central vision (Fig. 4).

### Crowding assessment 2: Polarity effects

#### Tasks 5 and 6: Same and reverse-polarity flankers

**Figure 5:**
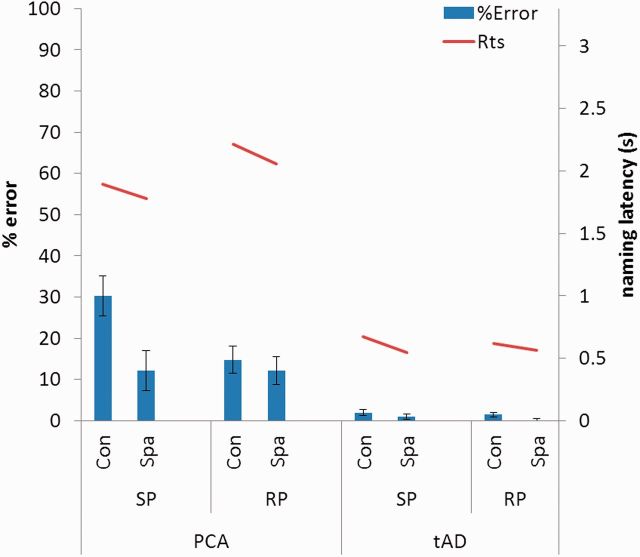
Accuracy and naming latency data for the PCA and typical Alzheimer’s disease (tAD) group for same and reverse polarity flankers in both spatial conditions. Rts = reaction time; Con = condensed; Spa = spaced; RP =reverse polarity ; SP = same polarity. Error bars show standard deviation.

A summary of the PCA and typical Alzheimer’s disease accuracy and naming latency data is shown in Fig. 5. The patients with PCA were consistently worse than both patients with typical Alzheimer’s disease and control subjects in terms of both naming accuracy and latency on Tasks 5–6. Differences in accuracy and latency between the typical Alzheimer’s disease and control groups did not reach formal levels of significance except for patients with typical Alzheimer’s disease being slower on Task 6.

#### Tasks 5 and 6: Effects of polarity

Patients with PCA (*n* = 22) showed significantly poorer accuracy for target letter identification with same rather than reverse-polarity flankers (z = −3.07, *P* < 0.005). This polarity effect only occurred for condensed flankers (condensed: z = 4.82, *P* < 0.001; spaced: *P* > 0.8). Analysis of latency data (*n* = 10) found a significant effect of spacing (t = 2.66, *P* < 0.05) but not polarity (*P* > 0.9) on naming speed. In the typical Alzheimer’s disease group, there was no significant effect of polarity on naming speed (*P* > 0.8) or accuracy (*P* > 0.1); however, there was an interaction between spacing and polarity (t = −2.63, *P* < 0.05), with condensed flankers of same polarity having longer naming latencies. Although none of the control subjects made any errors, there was a trend towards longer naming latencies with same polarity flankers (t = −2.18, *P* = 0.050), although there was no interaction between spacing and polarity (*P* > 0.1).

### Error analysis

The error responses in the PCA group (overall error rate: 17.7%) fell into three categories: (i) Type A (no response) in 29.9% of errors, the target remaining unidentified, which could result from participants being unable to either detect or identify the target; (ii) Type B (flanker identification), 36.3% error responses resulted from the participant providing the name of a flanker rather than the target (e.g. ZNH → Z); and (iii) Type C (letter not present in target/flanker array), in 33.8% error responses, the participant named a letter which was neither the target nor a flanker. The majority of these errors were suggestive of perceptual integration of flanker and target stimuli (YMT → V, 3T6 → C). This was despite accurate unflanked letter identification (overall error rate: 0.2%).

#### Averaging versus feature substitution

An error analysis was carried out in order to assess the extent to which averaging of target and flanker features (Pelli *et al.*, 2004; Greenwood *et al.*, 2009) or substitution of flanker features (Wolford, 1975) predicted the errors made by participants. Three models were constructed (Fig. 2), and applied to Type B and C errors (see above; total *n* = 358): (i) Model 1 (target/flanker averaging): similarity between the error response and the averaged target and flanker stimuli was a significant predictor of error rate (z = 13.40, *P* < 0.001); (ii) Model 2 (individual flankers): similarity between the error response and the individual flanker stimuli did not significantly predict error rate (right flanker: z = 1.68, *P* = 0.093; left flanker: *P* > 0.4; both: *P* > 0.5); and (iii) Model 3 (individual flankers and target): similarity between the error response and the target or individual flanker stimuli did not predict error rate (target: *P* > 0.4; right flanker: *P* > 0.1; left flanker: *P* > 0.4; both *P* > 0.7).

### Neuroimaging findings

**Figure 6:**
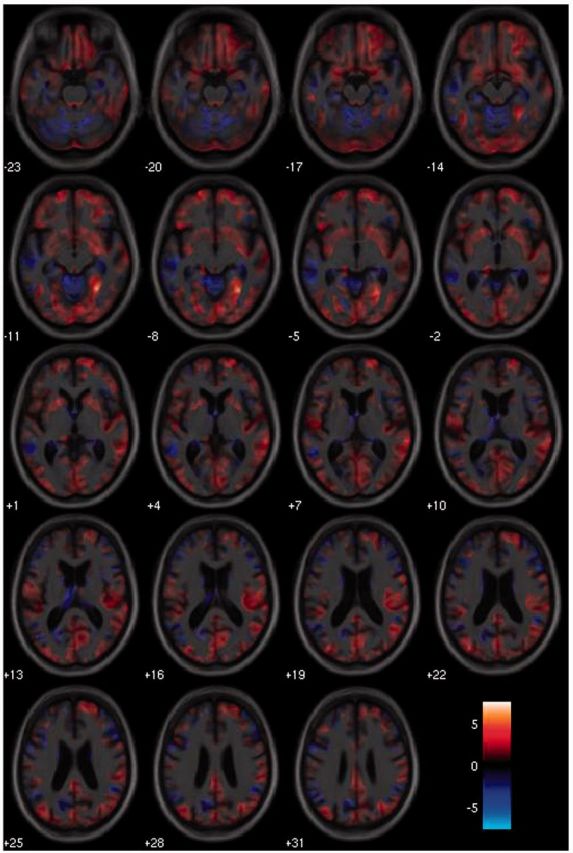
T-contrast effect maps showing associations between a measure of crowding [spacing (shapes/numbers)] and grey matter volume displayed on axial sections. Warmer colours indicate stronger positive associations between a greater degree of crowding and lower grey matter volume, with cooler colours representing the reverse contrast. The colour-map indicates t-values for this association.

**Figure 7:**
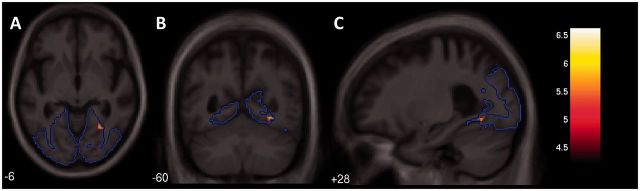
Statistical parametric map of grey matter volume associated with a measure of crowding [spacing (shapes/numbers)]. The SPM is displayed on axial (**A**), coronal (**B**) and sagittal (**C**) sections of the custom template in MNI space: the right hemisphere is shown on the right in coronal and axial sections. When restricting analysis to a prespecified region of interest (outlined in blue), there was an association between a greater degree of crowding and lower grey matter volume in the collateral sulcus (FWE corrected: *P* < 0.05; peak t = 6.61, location: *x* = 30 *y* = −58 *z* = −8): the colour-map indicates t-values for this association.

T-contrast whole brain effect maps showing neuroanatomical associations between performance on Tasks 3–4 and grey matter volume in the PCA group are displayed in Fig. 6. No significant associations between indices of crowding [crowding (shapes), crowding (numbers), crowding (shapes/numbers)] and grey matter volume were found when correcting for multiple comparisons over whole brain volume. When restricting analysis to the pre-specified occipital region, a significant negative correlation was found between crowding (shapes/numbers) and grey matter volume in the right collateral sulcus, between the fusiform and lingual gyri after correcting for multiple comparisons (*P* < 0.05): a more pronounced crowding effect for letters surrounded by shapes and numbers was associated with reduced grey matter volume in this region (Fig. 7). The location of the maximal association within this region lies at the boundary of the pre-specified occipital region; this, along with associations shown in the whole brain effect maps, suggest the critical region is not confined to the occipital lobe but extends into the temporal cortices. In Tasks 5–6, there were no significant associations between the discrepancy in accuracy between flankers of opposite polarity [polarity, polarity (condensed)] and grey matter over whole brain volume or within the pre-specified occipital region.

## Discussion

The study presents the first systematic investigation of crowding in a neurodegenerative disease population. In a series of centrally presented flanked letter identification tasks, the quality of patients with PCA performance was consistent with crowding in healthy or amblyopic individuals. Spacing, not flanker type, was the primary determinant of accuracy, and this spacing effect was significantly ameliorated when target letters were surrounded by reverse-polarity flankers. The PCA group were considerably slower and less accurate than the typical Alzheimer’s disease and the healthy control group in flanked letter identification tasks (Tasks 2–4) despite similar accuracy (and speed, relative to patients with typical Alzheimer’s disease) in naming letters presented in isolation (Task 1). This detrimental effect of closely-positioned flankers upon letter identification ability was associated with significant reductions in grey matter volume in the right collateral sulcus.

### Does the observed behaviour constitute (excessive) crowding?

Before considering the implications of this neurodegenerative perspective on crowding, it is first necessary to rule out various alternative explanations for the observed pattern of performance. First, the crowding effect in PCA could not be attributable to a generic ‘dementia effect’ as individuals with typical Alzheimer’s disease showed no difference compared to healthy controls in their accuracy on any of the flanked letter identification tasks, and estimates of disease severity could not account for the magnitude of the spacing effect. Second, attentional dyslexia is an unlikely candidate explanation as it involves deficits in the recognition of multiple, concurrently presented stimuli of the same category, for example letters presented with other letters (Warrington *et al.*, 1993; Humphreys and Mayall, 2001). In Tasks 2–4, spacing, not flanker type, consistently determined naming accuracy. While there was an interaction between spacing and flanker type, this may be a consequence of perceptual similarity, and hence crowding, as opposed to the category-specific deficit previously linked with attentional dyslexia. Third, it is doubtful that patients with PCA’s pattern of performance can be attributed to a deficit in peripheral spatial attention, one that might underlie a restriction in the effective visual field (Russell *et al.*, 2004, 2013). This deficit becomes apparent through the diminished recognition of large rather than small stimuli (Crutch, 2013; Yong *et al.*, 2014); however, the discrepancy in reading accuracy for large and small words did not account for the current findings. Fourth, similarly it is unlikely that simultanagnosia (a concept which relates closely to the notion of ‘restriction in the effective visual field’) can account for the observed phenomena, as simultanagnosia would be predicted to yield equally poor performance with reverse as same polarity flankers (given in both conditions the target and flankers are clearly perceptible and well within contrast acuity thresholds). Fifth, despite the grave visuospatial deficits often observed in PCA, there is little evidence for these findings being wholly accounted for by visuospatial impairment. Performance on tasks involving unflanked letter identification, early visual, visuoperceptual or visuospatial processing, digit span backwards or the A Cancellation task did not account for the spacing effect within the PCA group or the overall difference in accuracy between the PCA and typical Alzheimer's disease groups in Tasks 2–4. However, measures of visuospatial and visuoperceptual ability did account for the group difference in the spaced, but not condensed, condition. This suggests that poor accuracy in the spaced condition may largely reflect visuospatial and visuoperceptual impairment, with poor accuracy in the condensed condition primarily arising from crowding. Sixth, the normal ophthalmological assessments suggest that the pattern of performance on flanked letter tasks cannot be accounted for by poor acuity, macular or retinal abnormalities. Finally, seventh, the *post hoc* evaluation of fixation patterns during trials on which crowding errors were made indicate that at least these participants were using central vision, hence demonstrating that performance does not merely reflect normal peripheral crowding.

### Theoretical implications

The types of errors made by the patients with PCA have implications for accounts of crowding. Error responses were classified into three categories: no response (Type A), naming of a flanker (Type B) and responses not present in the target/flanker array (Type C). Different accounts of crowding would predict a differential weighting of errors towards these three types. Classic lateral masking (Polat and Sagi, 1993) would primarily predict no response (Type A) errors due to flankers preventing detection of the target stimuli. By contrast, substitution (Wolford, 1975; Krumhansl and Thomas, 1977) and integration/averaging accounts (Parkes *et al.*, 2001; Pelli *et al.*, 2004; Greenwood *et al.*, 2009) would primarily predict naming of a flanker (Type B) or a letter that combines features of the flanker(s) and target (Type C). Patients with PCA generated approximately equal numbers of Type A, B and C errors. This variation in error type is consistent with both a multi-stage view of crowding (crowding encompasses both detection and integration of features; Levi, 2008), and the relative heterogeneity in patterns of atrophy across individuals with PCA (variation in the pathological burden upon different cortical visual areas). All three types of errors are also observed in flanked item identification tasks in healthy peripheral vision (Strasburger, 2005).

Furthermore, in the current study we attempted not only to establish the frequency of error types on our central flanked letter identification task, but also to distinguish between averaging and substitution accounts. This was achieved by testing the visual similarity between individual error responses (Type B and C) and (i) the averaged image of the flankers and target (to test the averaging account); or (ii) the images of the individual flankers and target items (to test the substitution account). Analysis revealed that error identity was only significantly predicted by the averaged flanker/target images. These findings are most compatible with compulsory averaging accounts of crowding (Greenwood *et al.*, 2009) and suggest that PCA patient responses in our flanked letter identification task are at least partially accounted for by excessive pooling of target and flanker information. The finding of an association between error response and the averaged target/flanker image does not rule out a role for the substitution of flanker features or whole flankers, but does suggest such substitution accounts alone cannot explain the pattern of errors made within the PCA group.

In healthy vision, crowding has been interpreted as a pre-attentive process that uses averaging to regularize the noisy representation of (feature) position in the periphery (Greenwood *et al.*, 2009). Our data suggest that in patients with neurodegeneration of the occipital cortices, at least one component of the acquired excessive crowding observed can also be regarded as a pre-attentive process that uses averaging to regularize the pathologically noisy representation of feature information in central vision.

### Neural correlates of crowding

Relatively little is known about the neural correlates of crowding. Although the visual cortex has been implicated in most theories of crowding, a divergence of opinion occurs with more specific localization, ranging from V1 (Blake *et al.*, 2006), V2 (Chung *et al.*, 2007) and V4 (Motter, 2006; Liu *et al.*, 2009). A two-stage model of crowding including both feature detection and integration might involve V1 (Levi, 2008) and the extrastriate cortex (Robertson, 2003), respectively. Anderson *et al.* (2012) recently identified how crowded stimuli provoked increased functional MRI activation from areas V1–V4, also suggesting that crowding may be a multistage process.

Our imaging data suggest that, within the occipital region, scores indicative of prominent crowding effects were associated with lower grey matter volume within the right collateral sulcus, between the fusiform and lingual gyri. The maximal association within this region lay at the boundary of the occipital region, extending into temporal cortices. Without retinotopic mapping, it is difficult to be confident of the exact correspondence between anatomical location and visual area. However, in previous studies, similar regions have been classed as area V4 (Sereno *et al.*, 1995; DeYoe *et al.*, 1996; Hadjikhani *et al.*, 1998; Gallant *et al.*, 2000), V3 (Yeatman *et al.*, 2013) and V3a (Grill-Spector and Malach, 2004). Interestingly, V4 fulfils a variety of criteria that make it a promising locus for crowding. V4 is an area in which information from different stimulus types, orientations and spatial frequencies converge (Logothetis and Charles, 1990; Ferrera *et al.*, 1992, 1994; David *et al.*, 2006), receptive field size and anisotropy in V4 are similar in orientation and size to the radial/tangential anisotropy of crowding (Toet and Levi, 1992; Pinon *et al.*, 1998) and estimates of V4 receptive field size overlap with the extent of crowding in peripheral vision (Smith *et al.*, 2001; Chung *et al.*, 2007). Bias competition, in which patterns within receptive fields compete to determine the firing rate of individual neurons, has been localized in areas V4 and higher (Desimone and Duncan, 1995; Reynolds *et al.*, 1999; Chelazzi *et al.*, 2001); this may underlie crowding as a possible consequence of competitive feature integration processing (Nandy and Tjan, 2007). Anderson *et al.* (2012) cite how there is a significant increase in population-based receptive field size from V1 to V4 (Smith *et al.* 2001; Amano *et al.*, 2009), and suggest that crowding effects might accumulate from pooling of target and flanker stimuli over receptive fields of increasing size. Thus there appears to be a convergence between the current localization of the spacing effect in individuals with acquired excessive crowding and an array of normal human and animal data concerning neural correlates of feature averaging and integration.

The clear effect of polarity observed in these results (Tasks 5 and 6) might suggest which regions are implicated in at least one stage of crowding. Reverse polarity has been shown to segregate information via on and off pathways at the level of bipolar cells in the outer retina, which continue to stay relatively distinct until reaching the early visual cortex (Schiller, 1992). Although there is evidence of interaction between the two pathways (Wassle and Boycott, 1991; Harris and Parker, 1995), this segregation of information between target stimuli and flankers of reverse polarity may account for the observed alleviation of the crowding effect in our patients. Regarding the neural correlates of on and off pathway integration, Zhou *et al.* (2000) found that 48% of V1 and 20% V2 and V4 neurons in macaques encoded local contrast polarity, while the majority of neurons in V2 and V4 encoded direction-specific contrast polarity edges. Motoyoshi and Kingdom (2007) proposed a two stream model of second order processing, with the first stream composed of complex V1 cells sensitive to orientation and the second composed of lateral geniculate nucleus or V1 blob cells sensitive to polarity. However, similar temporal limits of the polarity advantage and attention (Chakravarthi and Cavanagh, 2007) contest the notion of this effect being an exclusively low-level process. Measures of this reverse polarity effect were not significantly associated with grey matter volume in our current data set, but this may reflect a simple power issue and replication with a larger patient cohort may enable us to discriminate between low- and high-level theories of the polarity effect.

Establishing a model of acquired excessive crowding in individuals with neurodegenerative disease may offer an opportunity to test mechanistic accounts of crowding through future pathological studies. For example, integration and averaging models of crowding (Parkes *et al.*, 2001; Greenwood *et al.*, 2009) propose that, while isolated contours are processed by simple cells, a high concentration of flanking contours in a small region or ‘integration field’ (Pelli *et al.*, 2004) leads to a greater response of complex cells which then suppress simple cell activity within their receptive field area. Applying this hypothesis to individuals with PCA, it is possible that simple cell activity in areas such as the primary visual cortex may be less disrupted by Alzheimer’s disease pathology than complex cells in more downstream visual areas, such as V4. Cells in V4 which suppress simple cell activity through connections with areas earlier in the visual system may be particularly susceptible to Alzheimer’s disease pathology: this would result in a diminished ability to suppress signals of high contour concentrations within an integration field. This proposal is at least consistent with previous neuroimaging (greater atrophy of lateral than medial occipital lobes; Lehmann *et al.*, 2011; Ridgway *et al.*, 2012) and histopathological reports in PCA (increasing density of neurofibrillary tangles and senile plaques from areas V1 to visual association areas: Hof *et al.*, 1997). Pathological studies have also pointed to the selective vulnerability of certain neurons to Alzheimer’s disease, particularly cells with long axonal projections (Lewis *et al.*, 1987; Morrison *et al.*, 1987) such as V1 Meynert cells in PCA (Hof *et al.*, 1990), and extensive disruption of feedforward and feedback projections by neurofibrillary tangles and senile plaques in Brodmann areas 17 to 19 (von Gunten *et al.*, 2006).

### Clinical implications, challenges and future directions

Both quantitative (spacing and polarity effects) and qualitative (error types) aspects of flanked letter identification performance, and the cortical localization of the spacing effect, indicate that individuals with PCA commonly develop excessive crowding in central vision. Just as crowding in the normal periphery operates irrespective of object type (e.g. faces: Martelli *et al.*, 2005; scenes: To *et al.*, 2011; see Pelli, 2008), so might acquired excessive crowding in the central vision of individuals with PCA and other neurodegenerative conditions have an impact upon a host of important everyday visually-guided behaviours and functions such as reading, face recognition or navigation. Studying crowding in neurodegenerative conditions such as PCA is not without its challenges; for example, the patients’ broader spatial cognition impairment has implications for stimulus selection and response modality (e.g. multi-feature letter stimuli were used in the current task as they can be verbally labelled, whereas Gabor patches or cross stimuli require manually or verbally-mediated spatial judgements about angle or spatial position). Nonetheless, complementing studies of the occurrence of crowding in structurally stable human models with studies of the emergence of crowding in degenerative models like PCA offers particular opportunities. In a neurodegenerative disease model of crowding, developments in high-resolution quantitative MRI which provide an *in vivo* window on cortical microstructure at the level of cortical layers, ocular dominance columns and stripes (Yacoub *et al.*, 2008; Sereno *et al.*, 2013), and amyloid plaques in Alzheimer’s disease (Meadowcroft *et al.*, 2009) can be exploited to improve understanding of the role of different cell populations in crowding. Examining the evolution of crowding in individuals with degenerating visual cortices (Yong *et al.*, submitted) will permit a novel, dynamic perspective upon the phenomenon and its interaction with basic and higher-order visual processes.

## Supplementary Material

Supplementary material

## Abbreviations

MMSE: Mini Mental State Examination
PCA: posterior cortical atrophy
