# Erratum

**DOI:** 10.1093/brain/awv033

**Published:** 2015-02-14

Keir X. X. Yong, Timothy J. Shakespeare, Dave Cash, Susie M. D. Henley, Jennifer M. Nicholas, Gerard R. Ridgway, Hannah L. Golden, Elizabeth K. Warrington, Amelia M. Carton, Diego Kaski, Jonathan M. Schott, Jason D. Warren, Sebastian J. Crutch. Prominent effects and neural correlates of visual crowding in a neurodegenerative disease population. Brain 2014; 137: 3284–99; doi:10.1093/brain/awu293.

This article has been corrected online and is available at brain.oxfordjournals.org.

The publishers would like to apologise for an error introduced during copyediting into the paper by Yong *et al.* On page 3286, the tasks listed under the heading ‘Crowding assessment’ should read as follows:

**Task 1: Unflanked letter identification**

The target stimuli (*n* = 20) were alphabetic items presented in isolation. Letters were presented in random order for 6000 ms in a fixation box (3.2° in width, 2.9° in height) at the centre of the screen.

In each of the following tasks (Tasks 2–6), target letter identification was probed under two spatial conditions, condensed and spaced.

**Task 2: Letter flankers**

Target letters (*n* = 24) were flanked on each side by a letter, forming a 3-letter non-word combination.

**Task 3: Shape flankers**

Target letters (*n* = 24) were flanked on each side by a triangle presented at different orientations. Triangles were of equal height and line thickness to target letters.

**Task 4: Number flankers**

Target letters (*n* = 24) were flanked on each side by an Arabic numeral, chosen from a range between 2 and 9.

**Task 5: Same-polarity flankers**

Target letters (*n* = 24) were flanked on each side by black letters; presentation was as Task 2 except that items were presented on a grey background to match Task 6 (see below).

**Task 6: Reverse-polarity flankers**

Target black letters (*n* = 24) were flanked on each side by white letters, all presented on a grey background.

