# Supplementary material for: Prominent effects and neural correlates of visual crowding in a neurodegenerative disease population
Source: Brain. 2014 Oct 28;137(12):3284–99. doi: 10.1093/brain/awu293 (PMC4240300; doi:10.1093/brain/awu293)
Supplement: Supplementary material [file brain_awu293_index.html]

Supplementary Data | Brain

## Supplementary Data

files

**Files in this Data Supplement:**

- Supplementary Data - docx file
