# Supplementary material for: Prominent effects and neural correlates of visual crowding in a neurodegenerative disease population
Source: Brain. 2014 Oct 28;137(12):3284–99. doi: 10.1093/brain/awu293 (PMC4240300; doi:10.1093/brain/awu293)
Supplement: Supplementary material [file e8e4c50b0a782459668f3dad55e3e85a_brain-2014-01004-File010.docx]

**Brain image acquisition**

T1-weighted volumetric magnetic resonance images were acquired on a Siemens Trio TIM 3T scanner (Siemens Medical Systems) for 20 PCA patients. Images were acquired using a 3D magnetization prepared rapid gradient echo (MP-RAGE) sequence producing 208 contiguous 1.1 mm thick sagittal slices with 28-cm field of view and a 256 × 256 acquisition matrix, giving approximately isotropic 1.1 mm cubic voxels; a 32-channel head coil was used.
